# Diffusion and spillover effects of an evidence-based mental health intervention among peers and caregivers of high risk youth in Sierra Leone: study protocol

**DOI:** 10.1186/s12888-020-02500-8

**Published:** 2020-02-27

**Authors:** Alethea Desrosiers, Praveen Kumar, Arja Dayal, Leslie Alex, Ali Akram, Theresa Betancourt

**Affiliations:** 1 Boston College School of Social Work, 140 Commonwealth Avenue, Chestnut Hill, MA 02467 USA; 2 Innovations for Poverty Action, 40 Wilkinson Road, Freetown, Sierra Leone

**Keywords:** Diffusion, Spillover effects, Implementation science, Youth, Low resource settings

## Abstract

**Background:**

Evidence-based mental health interventions have helped address health services gaps, but their reach and societal benefit can be limited in low resource settings. The current study extends an ongoing scale-up study of a cognitive behavioral therapy (CBT)-based intervention, the *Youth Readiness Intervention* (YRI), among high risk youth in post-conflict Sierra Leone to investigate mechanisms of diffusion and spillover effects of the YRI among peers and caregivers of youth who receive the intervention.

**Methods:**

We will recruit and enroll YRI index participants and control index participants (ages 18–30). Index participants will complete a standardized ego-network survey to nominate three peers in their social networks and identify their primary cohabitating caregiver. Identified peers and caregivers who consent to participate will complete a quantitative assessment battery on mental health outcomes, emotion regulation, and daily functioning at baseline and 8-month follow-up. Study outcomes also incorporate common indicators for implementation science, including measures of project context, evaluation, and scaleup. Social network analysis will investigate diffusion of YRI components across peer networks. Linear growth modeling will examine mental health spillover effects among caregivers. Incremental health costs and benefits among YRI participants’ caregivers and peers will be assessed through cost-effectiveness and return on investment analysis.

**Discussion:**

Assessing implementation research outcomes, including penetration of YRI effects across social networks and cost-effectiveness of the YRI as distinct outcomes, will provide key information about the success of YRI implementation. Lessons learned could inform decisions to increase scale up efforts in Sub-Saharan Africa and other low resource settings.

## Contributions to literature

- This study demonstrates: (a) the utility of including indirect health benefits of a cognitive behavioral therapy-based (CBT) intervention in return on investment analysis; and (b) the importance of accounting for naturalistic diffusion of CBT components to inform future implementation efforts in similar settings.
- This study amplifies inclusion of indirect effects into cost-effectiveness analysis, which can more accurately determine economic valuation and public health impact of mental-health interventions in post-conflict and low resource settings.
- This study explores components of a CBT-based intervention that are diffused across social networks of intervention recipients. Insights gained could address key sustainment challenges for future scale up strategies.
- This study illuminates diffusion pathways between intervention participants and their peers. Peer-led models for diffusion of CBT skills could be a cost effective strategy to extend the reach of evidence based mental health interventions in low resource and post-conflict settings.

## Background

Mental disorders are the second-largest contributor to the global burden of disease among youth and adults aged 14–55 and are projected to be the highest contributor of disability adjusted life years by 2030 [1, 2]. The treatment gap resulting from poor access to mental health services in nations with a history of violence and loss due to civil unrest, widespread disease (e.g., AIDS, Ebola), and other forms of adversity is well known [3, 4]. The prevalence of untreated mental disorders among adults in Low and Middle Income Countries (LMICs) may be as high as 78%; for adolescents and youth the figure is likely higher [5]. In settings where exposure to war and violence compounds other social problems, the risk of long-term mental health problems is also higher. Although feasible and effective evidence-based mental health interventions have closed health services gaps, their reach and societal benefits are often limited in low-resource settings.

The current study extends ongoing mental health services research in Sierra Leone on a cognitive behavioral therapy (CBT)-based intervention, the *Youth Readiness Intervention* (YRI). The YRI has demonstrated feasibility and effectiveness for improving emotion dysregulation, daily functioning, and educational outcomes [6–8], and methods for transitioning oversight to local partners and spreading evidence-based practices are currently being tested in Sierra Leone in partnership with the Government of Sierra Leone and the Deutsche Gesellschaft für Internationale Zusammenarbeit (GIZ) within an employment promotion program (EPP). The YRI’s demonstrated effectiveness in prior research [6–8] and its simple, transdiagnostic practice elements offer a highly suitable intervention for delivery in community settings by a range of lay workers in low resource settings with a basic level of education [8]. In this diffusion and spillover study, we will adopt a broader perspective to consider the indirect effects of the YRI on participants’ peer networks and cohabitating caregivers by examining (a) the extent to which indirect effects (i.e., diffusion and spillover) result in measurable incremental health outcomes in nonparticipants; (b) whether these indirect effects can be amplified in intervention design and implementation strategies; and (c) how to factor indirect effects into cost-effectiveness analysis to more accurately determine economic valuation and public health impact. Adopting a broader perspective may demonstrate the potential for the YRI and similar evidence-based mental health interventions in low resource settings to reach a larger population and result in both incremental mental health benefits and other societal benefits.

Understanding why and how core components of a transdiagnostic mental health interventions are diffused across social networks may address key implementation and sustainment challenges. *Diffusion* is the process of untargeted and unplanned spread of new practices over time among network members of a social system, including peer networks [9]. Implementation science frameworks often consider the process of diffusion but typically examine it at the provider level [10] rather than participant level. Recent pilot data indicates that YRI participants informally share the intervention’s CBT-techniques through peer networks [11]. Understanding the mechanisms through which evidence-based mental health interventions, such as the YRI, are naturally diffused among youth in low-resource settings can illuminate which elements of an intervention are most transferrable and help inform intervention design, delivery, and scale up decisions. 

Providing evidence of measurable spillover effects of mental health interventions in low resource settings has the potential to galvanize new investments in large-scale mental health initiatives. *Spillover* is the phenomenon of beneficial intervention effects to nonparticipants. Evidence-based mental health interventions delivered to youth may indirectly improve the mental health of other household members [12–14]. Preliminary data indicate that caregivers of youth receiving the YRI experience reduced emotional distress and burden of care compared with those of youth in the control condition [15]. We hypothesize that such health benefits occur because improvements in the mental and/or behavioral health of one youth might improve overall household dynamics; or because improved behavior of one youth might alleviate guilt or stress experienced by caregivers. If the YRI demonstrates a wider benefit (i.e., improving caregiver mental health), Sierra Leonean stakeholders may be further motivated to scaleup the intervention.

### Theoretical framework

YRI participants have reported that they enjoyed teaching friends techniques to improve emotion regulation, such as deep belly breathing and use of culturally relevant metaphors for emotion regulation (e.g., a pot of boiling water heats up before it reaches a boiling state). Rogers’ *Diffusion of Innovations Theory* defines diffusion as the process of communicating an innovation through specific channels over time among network members of a social system [9] and investigating the channels and patterns of communication within peer social networks. An innovation is “an idea, practice, or project that is perceived as new by an individual or unit of adoption [9].” Drawing from this theoretical framework, our study aims to investigate how youth receiving the YRI share and diffuse certain YRI components (CBT practices) with their peers and to determine which components are most transferrable. Youth may share components through knowledge transfer (e.g., modeling, demonstration) or persuasion (e.g., stories, sharing positive experiences). We will identify peer-to-peer diffusion (i.e., incidence, fidelity) of YRI components and investigate the degree to which this secondary delivery of the YRI is related to improvements in mental health (i.e., emotion regulation, functioning) in YRI participants’ peer networks. By demonstrating naturally occurring diffusion among peer networks; identifying how YRI participants share YRI components with their peers; and by identifying which social network measures are related to diffusion, we can inform strategies to facilitate reach and penetration of intervention effects.

Research on spillover effects of mental health interventions is sparse [15, 16], particularly in low-resource settings, where maximizing benefit and minimizing cost are essential [17]. Through our mixed-methods approach to investigating the mechanisms through which spillover effects occur and persist over time, we will generate insights that might be used to guide the development of a theoretical framework for mental health spillover effects in Sierra Leone and other low resource settings. For example, processes of diffusion could also be occurring in the household that result in mental health spillover effects among caregivers; or spillover effects might occur because the stress and burden of caregiving lessens as youth functioning improves. Key informant interviews with cohabitating caregivers, triangulated with quantitative data, will help to illuminate these potential pathways for mental health spillover.

Understanding and harnessing the mechanisms of diffusion and spillover occurring in the YRI may help address the challenge of delivering innovative interventions with sufficient breadth and depth to benefit large populations or large geographic areas in LMICs and other low-resource settings. By quantifying indirect mental health benefits occurring through diffusion and spillover, we will be able to examine cost-effectiveness from a broader societal perspective [18–20]—one that considers all health costs and benefits regardless of who incurs the cost or experiences the effects [21, 22]. A broader understanding of the societal value of evidence-based mental health interventions could influence policy, funding, and scaleup decisions.

#### Study objectives

The aims of the current study are to: (a) investigate the mechanisms of naturalistic diffusion of CBT components among YRI participant peer networks; (b) investigate the mental health spillover effects among YRI participants’ primary cohabitating caregivers in comparison to caregivers of youth in the control condition; and (c) conduct a return on investment (ROI) analysis in dollars and a cost-effectiveness analysis that factor measurable incremental health outcomes across peer networks and among caregivers of youth who receive the YRI.

We hypothesize that (a) the simplest to use YRI components (e.g., deep belly breathing, sequential problem solving) will be most typically shared among peer networks, whereas the more complicated techniques (e.g., cognitive restructuring) will be less shared; (b) YRI participants’ caregivers will report reduced sense of burden, improved mental health, and reduced functional impairment compared with caregivers of youth in the control condition; (c) the ROI and incremental health benefits of the YRI for participants and peer networks combined will be greater that the incremental health benefits for participants alone*.*

Our study aims to advance implementation science, as potential findings could influence design of mental health interventions targeting large populations in low-resource regions (e.g., incorporating elements to promote naturalistic diffusion and impact across social networks); how we measure implementation science outcomes (e.g., penetration); and how we assess the societal value of interventions. Given the limited overall health infrastructure in Sierra Leone and other low resource settings [23, 24], this is a unique opportunity to study how targeted mental health interventions may have incremental health effects beyond those receiving direct intervention support.

## Methods/design

### Overview of study design

Our proposed study will link to an ongoing NIMH-funded scale up study of the YRI delivered via the EPP among high risk youth in Sierra Leone (U19MH109989;) wherein a sub-sample of U19 participants will be recruited and enrolled as index participants in the current study. We will recruit and enroll 140 youth who received the YRI and 140 control youth from the U19-funded scale up study as index participants. YRI youth will have also received EPP training. Participant recruitment will take place in communities in 3 rural districts where the YRI is currently being scaled up: Kailahun, Kono, and Koinadugu. Staff will describe the study to U19 study participants and obtain informed consent to serve as index participants for the current study. All study procedures received Institutional Review Board and Sierra Leone Ethics and Scientific Review Committee approvals.

Peer social network data will be collected using an egocentric fixed choice study design, which samples a population of interest and collects information on each respondent and his/her contacts, resulting in a collection of egocentric networks [25]. At baseline assessment, we will administer a standardized ego network survey for enrolled U19 index participants (“egos” in the context of social networks) to generate a list of peers (“alters” in the context of social networks) and explore their corresponding tie strengths. We will collect social network data in a 3-step survey. First, our survey will begin with *name generator* questions to prompt identification of 3 peers aged 18 or older. Second, after eliciting peers, *name inter-relater* survey questions will evaluate the strength of connections (tie strength) between YRI participants (egos) and identified peers (alters). Third, *name interpreter* survey questions will solicit contact information (i.e., name, cell #, residence) and a range of characteristics of peers to aid in identification (e.g., nicknames, demographics, height, etc.). We will contact identified peers, verify that they know the U19 participant, and invite them to participate in the current study via informed consent.

Primary caregivers will be defined as the cohabitating household member primarily responsible for looking after the participant’s wellbeing. Prior to beginning the YRI, youth who consent to participate in the current study will be asked to provide information about their household composition, including number of individuals in the household, age and sex of all household members, relationship of each household member to the participant, and to identify their primary caregiver (i.e., name, contact information, relationship). If participants are unable to identify such a member, they will be asked to identify the person they are emotionally closest to in their household—under the assumption that this member would be the most invested in their wellbeing.

### Settings and participants

Inclusion criteria for peers are (a) be a part of a YRI participant’s social network; and (b) male or female aged 18 or older. Exclusion criteria are (a) be a current YRI participant; (b) severe, active suicidality or psychosis as assessed via the MINI-SCID diagnostic assessment administered by a study social worker; and (c) serious cognitive impairments that might preclude one’s ability to comprehend informed consent and complete study assessments as determined by a study social worker. Youth who report active suicidality or psychosis will be referred for immediate individual mental health services. If one or more of the YRI participants’ three identified peers do not consent to participate or is ineligible, we will recruit additional YRI participants, administer the standardized ego-network survey, and enroll their identified peers (alters) to reach our sample size. We will repeat this procedure to recruit control only peers.

Inclusion criteria for caregivers are (a) identified as a primary adult caregiver (aged 18 or older) of a YRI participant; and (b) residing in the household of a YRI participant. Exclusion criteria are (a) not residing in the household of a YRI participant; (b) severe, active suicidality or psychosis as assessed via the *MINI-SCID* administered by a study social worker; and (c) serious cognitive impairments that might preclude one’s ability to comprehend informed consent and complete study assessments as determined by a study social worker. Caregivers who report active suicidality or symptoms of psychosis requiring more intensive mental health care will be referred for immediate individual mental health services.

### Recruitment strategy

We will recruit and enroll 840 peers (*n* = 420 YRI peers; *n* = 420 control peers) and 280 cohabitating primary caregivers (*n* = 140 YRI caregivers; *n* = 140 control caregivers). A list of peers and caregivers will be elicited through the ego network survey that will be administered to U19 index participants (*n* = 140 YRI youth; *n* = 140 control youth).

#### Index participants

Index participants will be randomly sampled from the total sample of enrolled U19 study participants (*n* = 1200). Random sampling will be executed at the district level: Kailahun (*n* = 140, Kono (*n* = 70) and Koinadugu (*n* = 70). The number of youth sampled per district will be roughly proportional to the number of U19 participants in the respective districts.

Clusters in the U19 study were defined based on geographic and logistical considerations related to the implementation of the YRI and EPP (20 YRI + EPP clusters, 20 EPP-only clusters, and 20 no intervention control clusters). We will use a two-stage sampling strategy to select index participants. First, YRI + EPP and control clusters will be randomly selected at the district level. Then, we will randomly select an equal number of index participants from each cluster. The number of youth selected per cluster will be defined based on the district level recruitment targets described in the previous section.

#### Peers and caregivers

Detailed contact information for nominated peers and caregivers will be collected during administration of the ego network survey and used to locate peers and caregivers. We will employ a three-level tracking strategy to locate peers and caregivers. First, field team members will attempt to establish contact through mobile phone numbers listed by index participants. Multiple attempts will be made to establish contact over the phone. In cases where phone contact cannot be established, field team members will attempt to locate peers and caregivers in person based on the address listed by index participants. If an individual is not located at the listed address, field team members will attempt to contact the index participants to gather additional tracking information. If we are unable to locate a peer or caregiver through the three steps listed above, the entire procedure will be repeated within a period of 5 days. Individuals who cannot be located after four attempts of the above procedure will be classified as attrition.

### Measures

#### Peer outcomes

We will collect quantitative data on emotion regulation, daily functioning, prosocial attitudes, mental health, and risk behaviors among peers at baseline and 8-month follow-up (see Table 1). All quantitative measures underwent a thorough development, translation, and validation process in a prior randomized controlled trial for youth facing adversity in Sierra Leone and have demonstrated good reliability. The WHO process of translation and adaptation of instruments [26] was used to translate measures from English to Sierra Leonean Krio. Measures have been subjected to cognitive testing to ensure comprehension and cultural relevance in assessing satisfaction, social support, and fidelity as well as mental health, daily functioning, and other life outcomes. The following quantitative measures will be used in the current study: the Difficulties in Emotion Regulation Scale (DERS) [27] (α = .65); WHO Disability Assessment Schedule (WHODAS) [28] (α = .91); Brief COPE Scale [29] (α = .72–.84); the Conflict Tactics Scale [30] (α = .72–.86); and Inventory of Socially Supportive Behaviors [31] (α = .87); Hopkins Symptom Checklist [32] (α = .92); and the Adapted Youth Risk Behavior Survey [33] (α = .74–.86).

**Table 1:** Diffusion Study (Aim 1) Data Collection Methods for Peers

Construct/Indicator	Data Collection Method (Time point)	Instrument/Psychometrics/Means of Measurement
**Emotion Regulation; Daily Functioning;** **Coping Skills; Prosocial Attitudes;** **Anxiety, Depression & Risk Behavior** *Assess improvement in mental health and functioning in peers*	*Quantitative* • Survey (baseline, 8-mo)	• Difficulties in Emotion Regulation (DERS) • WHO Disability Assessment Schedule (WHODAS) • Brief COPE scale • Responses to Stress Questionnaire • Conflict Tactics Scale • Inventory of Socially Supportive Behaviors as used in Survey for War-Affected Youth in Uganda • Hopkins Symptom Checklist • Adapted Youth Risk Behavior Survey
**Process of Diffusion; Fidelity to YRI components**	*Qualitative* • Focus groups & dyad interviews	• Probes: what skills were shared? Why/how were they shared? • Fidelity rating scales • YRI Skills and Knowledge Questionnaire
**Core Metrics of Context; Evaluation; Scale-up; Penetration; Costs** *Assess implementation outcomes*	*Quantitative* • Survey (baseline, 8-mo)	• Daily Functioning scores (WHODAS) • Mental Health scores (WHO-5) • Cost-effectiveness analysis • Proportion in target population (peers) exposed to innovation • Frequency of contact with key stakeholders

### Caregiver quantitative outcomes

We will collect quantitative data on burden of care, daily functioning, coping, social support, emotion regulation, mental health, and quality of life among cohabitating caregivers at baseline and 8-month follow-up (see Table 2). The following measures will be used: the Difficulties in Emotion Regulation (DERS) [27] (α = .65); WHO Disability Assessment Schedule (WHODAS) [28] (α = .91); Brief COPE Scale [29] (α = .72–.84); the Conflict Tactics Scale [30] (α = .72–.86); Inventory of Socially Supportive Behaviors [31] (α = .87); Hopkins Symptom Checklist [32] (α = .92); and WHO Health-Related Quality of Life-Inventory (BREF) [34–36] (α = .85); Burden Assessment Scale [37] (α = .89–91).

**Table 2:** Spillover Study (Aim 2) Data Collection Methods for Caregivers

Construct/Indicator	Data Collection Method (Time point)	Instrument/Psychometrics/Means of Measurement
**Burden of Care; Daily Functioning; Coping;** **Social Support;** **Emotion Regulation;** **Anxiety, Depression; Quality of Life** *Assess reduced caregiver burden and improvement in mental health*	*Quantitative* • Survey (baseline, 8-mo)	• WHO Disability Assessment Schedule (WHO-DAS) • Brief COPE scale • Multidimensional Scale of Perceived Social Support; • Hopkins Symptom Checklist • Burden Assessment Scale • Conflict Tactics Scale • WHO Health Related Quality of Life-Inventory BREF • Difficulties in Emotion Regulation Scale (DERS)
**Process of Spillover and Experience of cohabitating primary caregivers**	*Qualitative* • Key informant interviews	• Probes: what was experience of having index participant in the YRI? How did this affect household members emotions and behaviors? How did this affect sense of burden?
**Core Metrics of Context; Evaluation; Scale-up; Penetration; Costs** *Assess implementation outcomes*	*Quantitative* • Survey (baseline, 8-mo)	• Daily Functioning scores (WHODAS) • Mental Health scores (WHO-5) • Cost-effectiveness analysis • Proportion in caregivers exposed to innovation • Frequency of contact with key stakeholders

### Implementation science outcomes

We will use common indicators of implementation science [38, 39] to allow comparison of findings to other contexts and advance implementation science across low-resource settings. We will assess penetration [38] of the YRI through the proportion of peers and caregivers directly and indirectly exposed to the YRI. We will assess cost through ROI and cost-effectiveness analysis. For scale up, we will broaden the number of individuals exposed to the innovation (peers, caregivers). For context, we will test a new strategy for future implementation efforts to include both the target population and the penetration of an intervention beyond the primary clients to their peers and families. We will also assess frequency of contact with key stakeholders.

### Quantitative data collection

YRI participants’ peers and cohabitating caregivers will complete quantitative assessments at baseline and 8-month follow-up. Trained research staff will administer assessments electronically using a tablet. To investigate diffusion of YRI effects, peers will complete the quantitative assessment battery to measure mental health, emotion regulation, prosocial behaviors, and daily functioning. To investigate mental health spillover effects, cohabitating caregivers will complete a similar quantitative assessment battery to measure mental health, daily functioning, and burden of care.

### Qualitative data collection

We will conduct active focus groups with 4 YRI index participants (2 males, 2 females) and 2 peers (1 male, 1 female) randomly selected from YRI sites at baseline (Post-intervention for YRI participants) and 8-month follow-up (12-month follow-up for YRI index participants). There will be four focus groups held at each time point. A research team member will guide group discussions in Krio, focusing on questions such as: *What components of the YRI have you shared (*e.g.*, deep belly breathing)? Who have you shared YRI components with? How did you share YRI components and skills (*e.g.*, demonstration, discussion)? How did sharing of YRI skills take place (*e.g.*, face to face, mobile device)?* To assess fidelity of peer-delivered components, we will ask focus group participants who report sharing YRI components with their peers to demonstrate (role play) how they shared. We will videotape the role-plays and use them to rate the fidelity of the YRI component with a fidelity checklist developed from the Youth FORWARD study tools. We will also conduct 12 dyadic interviews with pairs of YRI participants and their first-nominated peers (1 peer and 1 YRI participant per dyad) at baseline. Dyadic interviews will target the same topics areas as focus group discussions, but will focus more specifically on sharing and receiving of information within the dyad. Audio-recorded qualitative data, transcribed and translated into English with identifying information removed, will be analyzed using grounded theory [40, 41] and an analytical strategy derived from thematic content analysis [42]. We will subject all data to an open-coding process without prior assumption of a theoretical framework for diffusion. We will identify key themes and iteratively develop a coding scheme, organized according to key themes (e.g., sharing through demonstration). Two team members trained in coding scheme will independently code 10% of transcripts to examine reliability. Poor agreement (i.e., low kappa ratings) will be grounds for refining the codebook. Reliability testing will be repeated until all coding is at 80% reliability. Team members will code the full qualitative dataset in MaxQDA using the robust coding scheme [43]. YRI content knowledge will be assessed using a quantitative scale administered at the conclusion of focus groups and dyadic interviews.

We will conduct Key Informant Interviews with a sample of cohabitating caregivers of YRI participants to gain a deeper understanding of how spillover effects occur. Qualitative data will deepen our understanding of the mechanisms by which youth participation in the YRI may lead to reduced burden and improved mental health among cohabitating caregivers. We will conduct key informant interviews with 12 caregivers of YRI participants randomly selected from YRI sites (6 males; 6 females) at baseline (post-intervention for YRI index participants) and 8-month follow-up (12-month follow-up for YRI index participants). Trained research staff will guide interviews, with questions such as: *What, if any, change did you observe from their participation in the YRI? How did having them in the group affect how you felt? How did having them in the group affect your sense of caregiving burden?*

### Sample size and statistical power

Due to the egocentric study design, we have several small subgraphs, where each subgraph has the YRI participant youth (ego) at the “center” and the peers (alters) at the “periphery” forming what is often called a k-star in network analysis. Power was driven by the number of subgraphs (i.e., the number of ego-alter clusters). Plots for power versus standard effect size were generated for a social network size of 3 peers (alters) per youth receiving the YRI (egos). This totals 14 youth per YRI + EPP site (*n* = 140 total youth) and control sites (*n* = 140 total youth). Power was calculated for a 3-level hierarchical model with 3 peers clustered within index subject and index subjects clustered within site. At a power of .8 and an alpha of *p* < .05, we have the power to detect an effect size (standardized mean difference) in the range of .30, with an ICC of .03. Based on our earlier pilot study on caregiver spillover effects showing effects sizes from .31–.51 [15], a minimum detectable effect of .30 would be within range. Power considerations will allow us to generate insights on potential spillover effects and the range of implications they may have on cost-effectiveness. Using these same assumptions, the minimal detectable standardized effect size for caregivers (*n* = 280) is .41.

### Statistical analyses: peers

We will conduct social network analysis to investigate how intervention effects are diffused through peer networks to non-participating youth. We will examine key dimensions of social network structure: participation rate, representativeness, and social integration. *Participation rate* will be calculated from the proportion of YRI sessions attended by youth (egos). *Representativeness* will be determined by comparing average characteristics of YRI participants and their peers; representative YRI participants (egos) should be more similar to peers (alters) in behavioral and demographic characteristics than to external controls. Demographic characteristics will include gender and age; behavioral characteristics will include emotion regulation and conduct problems. We will investigate the extent to which alters are similar to the egos in terms of demographics and other individual-level characteristics. Because of homophily—the tendency of connected individuals to be similar to one another—we expect ties to occur more frequently between similar (e.g., same sex, similar age) vs. dissimilar (e.g., opposite sex, different age) pairs of individuals. We will examine to what extent pairwise homophily is associated with greater diffusion or adoption of YRI components. The network analysis will attempt to identify factors that are associated with the existence of ego-alter ties. We will use a simple logistic regression as a starting point to modeling. To model the effect of covariates, we propose to use bivariate differences of nodal covariates as predictors;

To investigate diffusion of mental health effects among YRI participant peer networks, we will use growth modeling (mixed models) to assess whether changes in peer emotion regulation, mental health, and daily functioning are associated with uptake of YRI components. We will fit a linear growth model to the data using either a standard 2-level model by incorporating a measurement model for outcome variables or expanding it to a 3-level model [44–46]. In a 2-level model, the 2 time points (baseline and 8-month follow-up) are clustered within persons at level 1, where time-varying predictors (e.g., uptake of YRI content) are also included, and alters are included at level 2, where time-invariant predictors associated with the subjects (e.g., demographics) are included. These models will be used to assess the degree to which mental health trajectories among peers are associated with uptake of YRI components.

### Statistical analyses: caregivers

We will use growth modeling to investigate mental health spillover effects among cohabitating caregivers. Growth modeling by means of multilevel modeling (mixed models) will be used to assess whether changes in caregivers’ sense of burden, functional impairment, and mental health over time are associated with their child/charge’s YRI participation. We will also compare changes in mental health and caregiver burden among YRI participants' cohabitating caregivers to changes among caregivers of youth in the control group. We also will examine whether changes in YRI participant mental health and functioning are associated with changes among cohabitating caregivers. With 2 time points, we will fit a linear growth model to the data using a standard 2-level model; the 2 time points are clustered within persons at level 1, where time-varying predictors (e.g., uptake of YRI skills) are also included. Level 2 is for caregivers, where time-invariant predictors (e.g., demographics) are included.

### Economic analysis

The ROI framework allows for the inclusion and comparison of multiple types of outcomes by using dollars as a common metric [47]. ROI is quantified as the ratio of the sum of all benefits minus the costs, divided by the costs (ROI = (benefits-costs)/costs). Our economic analysis will focus on the labor market performance of peers and caregivers of the YRI youth versus those of control youth. We will use a one-month recall period and a one-year recall period. Primary outcomes will be engagement (hours worked) and income, measured through both wage employment and self-employment/enterprise.

We will also conduct a cost-effectiveness analysis to estimate the societal cost per QALY gained. We will calculate the costs of delivering the YRI to youth in the U19 scale up study to estimate intervention costs, including salaries of staff, intervention site operating costs, and overhead costs related to the YRI. We will use an ingredients approach in which implementation activities are first identified, and then the financial and economic costs of carrying out these activities are quantified [48]. Financial costs account for actual monetary expenses while economic costs encompass all resources, including those volunteered, and opportunity costs incurred by participants. Activities include staff and interventionist trainings, participant recruitment, YRI session delivery, and review of work with the research team. We will calculate the estimated time invested by each YRI participant to derive a value of this time in dollars, based on median wages in Sierra Leone. We will get cost estimates from a range of sources (e.g., invoices, receipts); staff time will be full salary cost; clinical space costs will be estimated based on local rents; and all costs will be expressed in United States dollars.

The economic impacts of the YRI will be assessed for YRI participants and their peers and primary cohabitating caregivers, including improved mental health and labor market outcomes. Mental health effects of the YRI will be defined as the standardized mean difference for primary outcome measures between peers and caregivers of YRI beneficiaries and the control group. Labor market outcomes include income, hours worked, number of jobs held, job search effort and other work quality measures via standard questions added to the survey batteries for YRI youth participants control youth. The principal outputs of the ROI calculations will be total estimated monetary value of benefits (better health and increased productivity) [49] for peers and caregivers (separately and combined) relative to cost.

We will convert the WHO-DAS, a measure of functional impairment, to QALYs [50]. To generate QALYs from the WHO-DAS, we will calculate a summary score using the polytomous scoring algorithm for WHO-DAS scores at each time point. Summary scores will be related to population-based health state preferences to derive a utility score or disability weight (in the range 0–1) using population norms from the *WHO Multi-country Survey Study on Health and Health System Responsiveness* Nigeria data set. We will plot utility scores/disability weights across the duration of the study and derive the QALY difference between peers and caregivers of YRI participants and those of control participants—an approach validated in previous studies [50]. For the intervention to be deemed cost-effective, each QALY gained must cost less than three times Sierra Leone’s GDP per capita at purchasing power parity [51]; this follows standards for good value for money outlined in global health interventions [50, 52]. We will calculate incremental cost-effectiveness ratios for peers and caregivers (separately and combined) by examining differences in costs between YRI participants’ peers and caregivers and those of control participants in the denominator, and differences in the effectiveness expressed in QALYs in the numerator. Sensitivity analyses varying discount rates and costs will provide a range of estimates for incremental cost-effectiveness ratios.

### Study status

At the time of manuscript submission, the study was completing the baseline data collection phase. Table 3 presents information on the timeline of all study activities.

**Table 3:** Timeline and Milestones

	YR1 (2019/20)				YR2 (2020/21)				YR3 (2021/22)				YR4 (2022/23)				YR5 (2024/25)			
	1	2	3	4	1	2	3	4	1	2	3	4	1	2	3	4	1	2	3	4
Pre-test assessments and interview guides	**X**																			
Align peer and caregiver recruitment to U19 study	**X**																			
Peer and caregiver data collection from YRI sites		**X**																		
YRI peer and caregiver screening and recruitment		**X**																		
Peer, caregiver, and control group enrollment			**X**																	
Peer and caregiver baseline data collection				**X**																
Peer and caregiver 8-month data collection							**X**													
Cost-effectiveness and ROI data collection			**X**	**X**		**X**	**X**		**X**	**X**										
Preliminary data analysis									**X**	**X**	**X**									
Intensive data analysis completed												**X**	**X**	**X**						
Dissemination activities/meetings with stakeholders														**X**	**X**	**X**				
YRI dissemination tools/package of care refinement																	**X**	**X**	**X**	**X**

## Discussion

Potential challenges to executing the study design include difficulties identifying and/or tracking nominated peers and caregivers and recruiting across large geographical distances with poor infrastructure. In the rural areas of Sierra Leone, youth and families can be highly mobile, particularly to seek employment opportunities during certain times of the year (e.g., moving to farms during the planting/harvesting season). Due to this high level of mobility, contact information and residences of peers and caregivers might change between the time when this information is obtained from index participants and the time peers and caregivers are recruited. If all nominated peers and caregivers are not located, this will also impede our ability to reach our target sample size. Strategies for addressing this potential challenge include collecting highly detailed information about nominated peers and caregivers and recruiting additional index participants to nominate additional peers and/or caregivers.

Additionally, in rural areas where communities are very small, some youth might have difficulty identify three close friends with whom they share information. One approach to addressing this potential challenge is by training enumerators to continue probing youth until the target number of peers are identified. Similarly, different youth could nominate the same peer in small or tight-knit communities. We will develop detailed tracking systems to identify such cases and engage in problem-solving discussions as a team to decide the best approach to addressing these cases during different phases of the study (e.g., recruitment, data collection, data analysis).

Despite these potential challenges, our study addresses important gaps in the research on implementation of evidence-based mental health interventions in LMICs and other low resource settings. While there have been significant scientific advances in development and evaluation of evidence-based interventions in these settings, systematic investigations to assess their indirect effects are scarce. This limits comprehensive inference on outcomes of mental health interventions. We bridge this gap in the current study by: (a) exploring the naturalistic diffusion and spillover of YRI components; and (b) analyzing the return on investment of the YRI by considering indirect benefits experienced by YRI participants’ peers and caregivers. This study will determine diffusion pathways of YRI components, which YRI components are most likely to be transferred from YRI participants to peers, the degree to which fidelity to the YRI component is demonstrated in peer delivery, and the effects of adoption of these practices among peers.

Systematic understanding of diffusion effects and holistic analyses of ROI by factoring in indirect effects of mental health interventions could be leveraged for future scale up strategies. Lessons learned can enhance the dissemination and implementation of evidence-based mental health interventions. For example, understanding pathways of diffusion through YRI participants and their peer networks could inform development of peer-led models, which could be a cost-effective alternative for delivering evidence-based behavioral interventions for vulnerable youth in low-resource settings. A better understanding of information sharing processes in youth social networks could improve development and implementation of feasible, effective, and sustainable delivery models [53–56].

## Data Availability

Not Applicable.

## Abbreviations

CBT: Cognitive Behavioral Therapy
DERS: Difficulties in Emotion Regulation Scale
EPP: Employment Promotion Program
GDP: Gross Domestic Product
LMIC: Lower-and Middle-Income Country
QALY: Quality-Adjusted Life Years
ROI: Return on Investment
WHO: World Health Organization
WHO-DAS: World Health Organization Disability Assessment Schedule
YRI: Youth Readiness Intervention
